# Could Amyloid-β 1–42 or α-Synuclein Interact Directly with Mitochondrial DNA? A Hypothesis

**DOI:** 10.1021/acschemneuro.2c00512

**Published:** 2022-09-20

**Authors:** Duygu Gezen-Ak, Zuhal Yurttaş, Tugay Çamoǧlu, Erdinç Dursun

**Affiliations:** Department of Neuroscience, Institute of Neurological Sciences, Istanbul University-Cerrahpasa, 34098 Istanbul, Turkey

**Keywords:** Mitochondrial DNA, mtDNA transcription, amyloid β, α-synuclein, neurodegeneration, mitochondrial dysfunction, Alzheimer’s disease, Parkinson disease

## Abstract

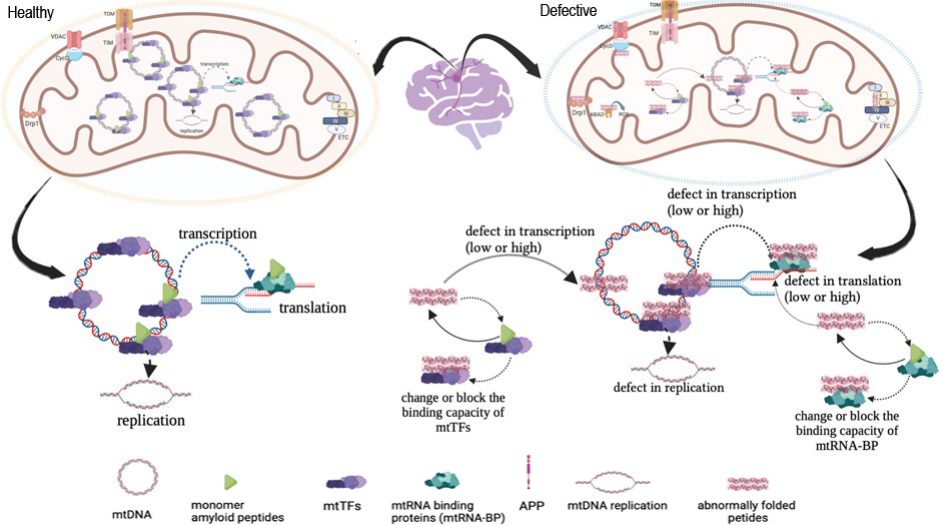

The amyloid β (Aβ) and the α-synuclein (α-syn) are shown to be translocated into mitochondria. Even though their roles are widely investigated in pathological conditions, information on the presence and functions of Aβ and α-syn in mitochondria in endogenous levels is somewhat limited. We hypothesized that endogenous Aβ fragments or α-syn could interact with mitochondrial DNA (mtDNA) directly or influence RNAs or transcription factors in mitochondria and change the mtDNA transcription profile. In this review, we summarized clues of these possible interactions.

## Introduction

Alzheimer’s disease (AD), which is the most frequently seen neurodegenerative disorder, is characterized by the accumulation of hyperphosphorylated microtubule-associated tau protein as intraneural neurofibrillary tangles and by the accumulation of amyloid-β peptide as extracellular amyloid plaques in the brain.^1^ On the other hand, Parkinson’s disease (PD) is defined by Lewy bodies and Lewy neurites, which are dominantly composed of α-synuclein (α-syn) protein.^2,3^ In both diseases, defects in energy metabolism due to mitochondrial dysfunctions occur during the neurodegeneration process. Mitochondrial dysfunctions are suggested to be common processes in neurodegenerative diseases such as Parkinson’s disease, Alzheimer’s disease, Huntington’s disease, and amyotrophic lateral sclerosis.^4^ The most well-known feature of mitochondrial dysfunction is increased reactive oxygen species (ROS) due to impaired oxidative phosphorylation (OXPHOS).^5^

Both amyloid-β and α-syn are classified as intrinsically disordered proteins and known to play critical roles in many cellular functions including DNA and RNA binding,^6^ and their aggregations are known to contribute to related diseases’ pathologies by increasing oxidative damage and disruption of cell membrane integrity.^7,8^ Because Aβ1–42 is produced from the cleavage of amyloid precursor protein (APP) located on the plasma membrane, it is considered an extracellular peptide. However, Aβ1–42 was demonstrated to be produced inside the cells or uptake into the cell from the extracellular space.^9,10^ Aβ1–42 is known to be involved in nonpathological conditions such as synaptic activity, neuronal survival, ion channel formation, and cholesterol transport regulation. Yet, these tasks have not been sufficiently understood.^11,12^ These findings support the importance of understanding the intracellular activities of the Aβ1–42 peptide.

On the other hand, although there is no detailed description of its function, it is thought that α-syn has roles mainly in the synaptic region and dopamine vesicle regulation.^13,14^ In addition to its functions in the synaptic field, it has been reported that α-syn interacts with many other cellular components such as mitochondrial proteins TOM40 and TOM20,^15,16^ RNA molecules,^17^ and histones.^8^ Besides, its role in endoplasmic reticulum-Golgi traffic^18,19^ and transport of microtubules^20^ is also reported.

Mitochondrial dysfunctions in energy metabolism are considered one of AD’s early hallmarks.^4,5,21^ It is pointed out that increasing Aβ1–42 causes accumulated damage in mitochondria, which induces cognitive decline by triggering neuronal dysfunction before the clinical onset of AD.^21^ It has been noted that Aβ fragments can be transported to mitochondria and localized there and form aggregates under pathological conditions.^22^ The finding that Aβ1–42 is localized in mitochondria has revealed new research areas about its goals and functions in mitochondria. Aβ1–42 plaque density was increased in the transgenic AD mouse model (APP/Ld) which carries mutation that inactivates the proofreading function of mitochondrial DNA polymerase γ (PolgA D257A).^23^ Aβ1–42 is suggested to interact with Drp1, cyclophilin D (CypD), cytochrome *c* oxidase, VDAC, and Aβ binding alcohol dehydrogenase (ABAD) proteins in mitochondria.^24^ Before the clinical diagnosis of AD, in the early stages, it has been reported that many of the nuclear genes encoding OXPHOS subunits had decreased expressions in the brains of those who suffer from mild cognitive impairment (MCI).^25^ Remarkably, in one of the studies with AD and MCI patients, it was reported that the expressions of some OXPHOS genes encoded by mtDNA varied according to the control group.^26^

Furthermore, Aβ1–42 can translocate into the nucleus, bind to nuclear DNA, and regulate the expression of some genes. Ohyagi et al. demonstrated that intracellular Aβ1–42 could induce p53 expression by binding to a known heat shock element located in the promoter region of the TP53 gene.^27^ Recently, a study indicated that Aβ1–42 could bind to DNA from its N-terminal region.^28^ Besides, critical research showed that Aβ1–42 could bind to promoter regions of specific genes that induce its production through an interacting domain.^11,29^ Our previous study also demonstrated the alterations in the expression of neurodegeneration-related genes due to Aβ1–42 presence.^9^ Such evidence led us to think that one of the targets of amyloid-β in mitochondria could be mtDNA.

Similar to the AD research results, increasing evidence draws attention to a relationship between the PD process and mitochondrial defects, all of which cannot be a coincidence. The mitochondrial quality control genes PINK1, PARKIN, LRRK2, and DJ1, whose mutations lead to familial PD, are examples of this relationship.^30−33^ Even though a substantial part of studies searches for the role of α-syn aggregation in the PD formation and progress, the relationship between these genes and the disease pathogenesis has not been fully elucidated yet. One of the most known examples is that 1-methyl-4-phenyl-1,2,3,6-tetrahydropyridine (MPTP) leads PD in humans and primates.^34^ This chemical shows its effect by blocking the OXPHOS complex I function.^35^ Like MPTP, it was reported that α-syn affects the same complex in PD brains.^36^

α-Syn can be located in the outer mitochondrial membrane^37,38^ and inner mitochondrial membrane.^36^ Also, increased levels of α-syn in the mitochondrial membrane and reduced activity of the mitochondrial complex I in the PD brains were reported.^36^ However, the most striking finding for us is that α-syn can be found in the mitochondrial matrix of neurons, too.^39,40^ This finding is prominent to know that α-syn can bind to nuclear DNA and even participate in the expression of some genes.^41^ α-Syn can enter the cell nucleus,^42^ binds directly to DNA, particularly to GC-box regions,^43,44^ and modulates DNA repair.^45^ Supporting the findings above, recently, we showed that some of the mtDNA-encoded genes and mitochondrial quality control genes (PARKIN and PINK1) expression are altered depending on age in sporadic and familial PD cases compared to the healthy controls.^46^

Mitochondrial dysfunction, alteration of the expression levels of proteins in mitochondria, and OXPHOS complex activity change with aging are reported in a normal physiological process. Especially the brain is one of the organs most prone to damage in the aging process due to its high energy requirement.^22,47,48^ However, neurodegenerative diseases are not seen in every aging individual worldwide. This means that brain cells can somehow tolerate the decline in mitochondrial functions. On the other hand, the main pathological components that play a role in neurodegenerative diseases seem somehow to eliminate this tolerance of brain cells. From another point of view, the normal physiological role of peptides such as Aβ or α-syn may be to regulate mitochondrial functions. The intermediates formed by the abnormal folding of these peptides may prevent them from performing their normal physiological functions, leading to increased mitochondrial dysfunction. If Aβ or α-syn can bind to mtDNA as a normal physiological function, they may be involved in many steps from transcription to replication. In this case, the pathological forms of these peptides may also have a direct role in increasing mtDNA somatic mutations, especially in the brain regions associated with the disease. For example, high levels of mtDNA point mutations or deletions were reported in the substantia nigra of PD patients^49^ and in the frontal cortex and hippocampus of AD patients.^50^ Elevated levels of mtDNA control region point or other mutations in AD brain were reported. These studies suggested that the mutations resulted in disruption of mtDNA transcription and triggered the replication errors.^50−52^ Although some studies did not find any change in mtDNA copy number^47^ in brain regions of patients suffering from PD or AD, others reported decreased mtDNA copy number in substantia nigra and cerebrospinal fluid (CSF) of PD patients^53−56^ or in the frontal cortex, hippocampus, cerebellar cortex, and CSF of AD patients.^57−59^ mtDNA copy number attenuation results can be interpreted as a decrease resulting from neuronal death. It can also be interpreted as the inability of these peptides, which function in replication under normal physiological conditions, to perform their functions due to their aggregation in pathological conditions. Looking at the mitochondrial cascade hypothesis, which discusses AD as a result of mitochondrial dysfunction, from another angle, the genetic background that determines the baseline functions of mitochondria and inherited by mtDNA and nDNA may be functioning properly in the presence of peptides such as Aβ or α-syn. Loss of function of these peptides with abnormal folding may change the disease’s formation, course, and severity with the contribution of genetic background. If changes in the genetic background have such a determinant, alterations in mtDNA replication and transcription may contribute to the neurodegenerative disorders and can be monitored systemically. One of the best proof of this is cytoplasmic hybrids studies with mtDNAs obtained from platelets of AD patients.^60^

Previous studies pointed out that α-syn, Aβ1–42, and prion proteins can bind to DNA, and this was suggested as a common feature of such peptides.^10,41,61^ As we mentioned above, both Aβ and α-syn localized in the mitochondrial matrix. These molecules also have nuclear DNA binding capacity. There has been no study focused on the possible binding of Aβ1–42 or its fragments and α-syn to mtDNA yet. Given these findings, we hypothesize that α-syn and Aβ1–42 may interact with mtDNA or mtDNA interacting proteins like TFAM under the physiological conditions and may change the mitochondrial gene expression pattern. We also consider the possibility of these peptides changing the expression pattern by binding mitochondrial RNA or transcription factors (TFs).

## Could Aβ1–42 or α-Syn Bind to mtDNA?

Our group and other researchers reported that Aβ1–42 could be found in the nucleus, and nuclear membrane pores can allow direct transport of 4 kDa Aβ into the nucleus. Aβ can bind to the region “KGGRKTGGGG”, a common sequence in APP, BACE1, and APOE promoters, and change the expression of many genes^11,29,62−64^ On the other hand, our group has shown for the first time in the literature that Aβ1–42 migrates from the cytoplasm to the nucleus in response to different antibiotic doses in primary cortical neurons.^62^ In the absence of antibiotics, we observed that Aβ1–42 was found in the nucleus but was more localized in the cytoplasm and translocated toward the nucleus as the antibiotic dose increased.^62^ These coincidental results may indicate that Aβ1–42 can bind DNA to a large extent and act like a gene regulatory protein or a TF. In particular, Barucker et al. showed that Aβ1–42 also plays a role in gene suppression by binding to LRP1 and KAI1 promoters.^64^

But why are we questioning whether it can bind to mtDNA? The latest data we have obtained about α-syn and a few studies that have observed similar results in patients with Alzheimer’s disease (AD) and mild cognitive impairment (MCI) lead us to this point. No such question occurred to us until we saw the clear pattern of genes encoded by mtDNA in Parkinson’s patients. In that study, we classified Parkinson’s patients primarily according to age, family history, and clinical characteristics. Then, we followed the expression levels of 13 oxidative phosphorylation (OXPHOS) genes encoded by mtDNA and PARKIN and PINK1 encoded by nuclear DNA. Also, we determined the amount of intracellular ATP levels of PBMCs of the patients.^46^ The data that we gathered were separating the patient groups from each other and healthy individuals. This pattern showed us that there was a difference in the expression of genes encoded by mtDNA, especially in sporadic patients. This difference was not a decrease but an increase in expression contrary to what was expected. We did not expect the increase in expression of mtDNA genes because almost all studies in the literature showed that OXPHOS activity decreased with neurodegeneration.^65,66^ Except for a few studies, the expression of mtDNA was ignored because everyone focused on activity. Noureddine et al. investigated the expression of mtDNA encoded genes in substantia nigra tissues of Parkinson’s patients, showed a similar increase in expression, and reported that this increase in transcription is not associated with the increase in the mitochondrial genome. They made the following explanation: “Increased mtDNA expression in substantia nigra of Parkinson’s patients may be due to high rates of transcribed mitochondrial genome or the half-lives of transcripts being longer than normal.”^67^ By the way, their results are almost entirely consistent with the results we found in leukocytes. What is interesting here is that we have obtained very similar results from leukocytes of PD patients, which have significantly short lifespan to be compared with neurons. On the other hand, we know that mitochondrial function is vital for postmitotic cells with high energy needs. For example, studies are suggesting that the risk of heart attack is increased in patients with PD and may be considered a nonmotor symptom.^68,69^ All this information shows that alterations in pathways related to mtDNA transcription may be systemic as well as resulting in OXPHOS dysfunction.^70^ Besides, we know that α-syn is expressed in many tissues, including blood.^71,72^ Many different models demonstrated that it is localized in mitochondria (inner–outer membrane, matrix) and can disrupt mitochondrial function by interacting with OXPHOS complex I,^36−39,73,74^ but it has never been investigated that it may be related to mtDNA or mtDNA transcription. There is also evidence that blood levels of α-syn increase with disease progression in Parkinson’s patients.^71^ In light of all this information, we hypothesize that abnormalities or disruptions in the transcriptional regulators involved in mitochondrial homeostasis in sporadic Parkinson’s patients may somehow cause expression changes, and one of these transcriptional regulators may be α-syn. It is known that α-syn acts as a transcription factor, binds to nDNA, and alters the expression of many genes.^41^ In this case, it may also bind to mtDNA. Mitochondrial dysfunction is among the basic neurodegeneration mechanisms not only for PD but also for AD. Cytochrome *c* oxidase-induced mitochondrial dysfunctions have been shown in the development and progression of AD. Data show that Aβ fragments are localized in the mitochondria and that their toxicity impairs and weakens mitochondrial functions.^65^ A critical study here is that Lunnon et al. showed the expression of genes encoded by mtDNA in the leukocytes of AD and MCI patients increased, just as we found in PD patients.^75^ However, the most notable difference between Lunnon and our study is that some of the genes with increased expression are different. The results show a difference between AD and PD in terms of complex I and complex III. So why does this difference arise? Although related studies show that pathological peptides can interact with complexes, another possibility is that Aβ1–42, whose function is impaired in AD, and α-syn, whose function is impaired in PD, can bind to mtDNA like a TF, causing the formation of different mtDNA expression profiles.

So, are there any findings that other proteins, other than the few known mitochondrial transcription factors, can bind to mtDNA? The mitochondrion has an evolutionarily conserved prokaryotic-like system that separates it from the rest of the cell, with its independent genome, polycistronic transcripts, and mitochondrial transcriptional regulation. However, mtDNA transcription depends mostly on mitochondria specific factors encoded in the nDNA. mtDNA transcription is driven by mitochondrial transcription factor A (TFAM), mitochondrial transcription factor B2 (TFB2M), mitochondrial RNA polymerase (POLRMT), mitochondrial transcription elongation factor (TEFM), and mitochondrial transcription termination factor (MTER).^76,77^

Although mtDNA’s transcription system preserves some ancient prokaryotic features like polycistronic mtDNA transcripts, some mitochondrial factors and nuclear transcription factors were demonstrated to bind to mtDNA and directly regulate its transcription. Potentially, nuclear transcription factors (TFs) can take part in mitochondrial gene expression in two ways, directly or indirectly. The arrangement of nuclear TF’s expressions of mitochondrial genes encoded in the nuclear DNA is called indirect regulation. These gene products are proteins or mitochondrial TFs that participate in the structure of mitochondria and take part in bioenergetic functions. Nuclear respiratory factors 1 and 2 (NRF-1 and NRF-2) can be shown as examples of TFs that take part indirectly. On the way to direct regulation, nuclear TFs can migrate to mitochondria and directly regulate mitochondrial gene expression.^78−80^ The first evidence for such an arrangement emerged with the determination of thyroid hormone action. This hormone binds to the D-loop and the 12S rRNA gene and alters mitochondrial gene expression when administered to nucleus-removed cells.^81^ Thus, it has been proven that nuclear TFs can directly take part in mitochondrial gene expression regulation, and this process can be performed independently of the nucleus. T3 receptor p43, CREB, p53, Stat3, estrogen receptor (ER), nuclear factor-κβ (NFκβ) TF family members, and glucocorticoid receptor (GR) can all be cited as examples of the best-defined nuclear TFs in mammals, involving mitochondrial gene expression.^82−85^ c-Jun, JunD, and CEBP β in human cells were shown to bind mtDNA^86^ The members of the NFκβ TF family are localized to mitochondria. RELA, a member of this family, binds to the mtDNA D-loop region in human cells in the absence of p53, reducing the levels of CO3 and CytB mRNAs encoded by mtDNA.^84^ In most of these studies, it was assumed that the TFs bind to target sequences on the D-loop region containing only the most well-known mtDNA transcription regulatory elements. However, D-loop binding does not apply to c-Jun, JunD, and MEF2D and under some circumstances to CEBPB, which bind to sequences in genes encoded in mtDNA. In summary, it is suggested that just like the exons of some genes in the nuclear genome can act as transcriptional regulatory elements of other irrelevant genes, a similar logic may also apply to mtDNA.^87^ Extensive research conducted by Mercer et al. on the mitochondrial transcriptome has shown that 159 DNaseI footprints cover 8.4% of the mitochondrial genome. These results indicate that there may be DNA–protein interaction points in mtDNA other than known ones.^87^ Besides, it has been reported that variations in these regions may play a role in the emergence of some diseases such as cardiomyopathy and type 2 diabetes.^88,89^ This information has led us to think that Aβ1–42 or α-syn, which can bind to promoters in nDNA like a TF, can bind to mtDNA like the TFs exemplified above.

## Could Aβ1–42 or α-Syn Bind to Mitochondrial RNAs?

Although the origin of mitochondrial RNAs is polycistronic, studies have reported that transcription of mature mRNAs can be managed by many different post-transcriptional mechanisms and show high variability.^87^ Studies show that α-syn easily binds to its own mRNA, prevents overexpression, and supports the optimal protein expression level.^17^ Investigating the role of RNA–protein interaction in the pathogenesis of human diseases has attracted considerable attention in recent years. This approach is thought to offer a new, up-to-date tool for the development of RNA aptamers, a useful therapeutic tool for the detection and control of neurodegenerative diseases.^41,90^ There is a growing view that the association of α-syn with RNA and other protein–RNA interactions plays a role in PD and other neurodegenerative diseases.^91,92^ One study identified 225 proteins that interact with α-syn in living α-syn treated neurons. This study showed the interaction of α-syn with proteins involved in synaptic transmission, endocytosis, and mRNA metabolism (RNA binding, processing, and translation factors). For example, polyadenylate-binding protein 1 (PABPC1) is an mRNA binding protein that facilitates the transport, destruction, translation, and stability of mRNA out of the nucleus and has been determined to interact with α-syn. Researchers suggested that α-syn can bind to the 3-poly(A) tail of mRNA, participate in polyA shortening, physically interact with translation factors, and play a role in the initiation of translation.^93^ This and other information link α-syn metabolism to mRNA metabolism, translation, and vesicle trafficking and thus Parkinsonism and neurodegenerative disease risk factors through molecular pathways to α-syn toxicity.^41^

When we searched the literature for Aβ1–42, we could not find any data showing that this peptide directly interacts with any known RNA, except for a few hypotheses and simulations. However, as we explained in the section above, we know that it interacts with nDNA.

It has been known for many years that amyloids can interact with metal ions and that Aβ precipitates with iron, zinc, and copper ions. Simulation studies show that Aβ1–42 has an α-helix folding in monomer form, and β-sheet folding can start with zinc binding.^94^ Zinc binding is not only related to the aggregation process but also can stimulate nucleic acid binding. Khmeleva et al. showed that zinc ions significantly enhance the binding of RNA and DNA molecules to Aβ1–42 aggregates.^28^ It is speculated that the binding of the zinc ions to Aβ aggregates can cause it to acquire a trait like the zinc finger transcription factors. In a study conducted in 2016, it was shown that Aβ16 (the region of Aβ that interacts with metals) could interact with RNA by using synthetic, randomly generated DNA and RNA molecules.^95^ In 2017, a simulation study performed using prion proteins with amyloid properties and three different miRNA sequences as a model showed that specific regions of amyloid oligomers could interact with RNAs.^96^

## Could Aβ1–42 or α-Syn Interact with Transcription Factors Found in Mitochondria?

It is suggested that TFs, which regulate the expression of genes encoded from nDNA, can function by binding to mtDNA, as described earlier. We think that Aβ1–42 (and/or Aβ1–40) and α-syn have this capacity due to the information described above.

The strong but complex results we obtained while investigating the effects of vitamin D receptor (VDR) on the production of Aβ1–42 in our previous studies^62^ showed that we could not explain the relationship between these two just because VDR is a TF. We hypothesized that VDR could be localized in the neuron plasma membrane with proteins involved in the production of Aβ1–42 and could involve its processing. To validate this hypothesis, we first had to show the presence of VDR in the membranes of neurons, and we demonstrated its presence in neuron membranes for the first time in the literature with live-cell surface staining experiments.^97^ In this article, we also determined that VDR is at least in certain proximity with APP, ADAM10, and nicastrin by immunofluorescent labeling. However, to support the hypothesis that VDR could coexist with proteins involved in the production of Aβ1–42 in the membrane, we had to hypothetically demonstrate the possible existence of a scaffold that could hold these proteins together. For this reason, we used the FpClass protein–protein interaction (PPI) prediction program to scan 5244 protein partners and create a possible placement prediction in the membrane.^97,98^ During this process, we have seen that the APP, precursor protein of Aβ1–42, can interact with TFs according to FpClass data.^97^ Regardless of the VDR, we reanalyzed all TFs that the APP could have a relationship with, we found over a hundred TFs, and we determined that 57 of them scored above 0.5 and 23 of them scored above 0.7. Again, we used the FpClass PPI prediction program for α-syn, and we found that they could associate with a total of 562 proteins. 21 of them with a score higher than 0.5 were TF, and 8 of these TF’s scores were higher than 0.7. Putting aside the ability to bind to DNA and starting from the knowledge that TFs regulate gene transcription by interacting with other TFs or proteins, these data showed us that Aβ1–42 (and/or Aβ1–40 or other fragments) or α-syn could also interact with TFs and effect transcription indirectly. On the other hand, since FpClass has an accuracy rate of 40% for proteins with high (score of >0.7) and moderate (score of >0.5) confidence intervals,^99^ the probability of Aβ fragments and α-syn to be associated with TF was quite high. While there is no study in the literature regarding Aβ1–42 and TFs relation, studies indicated that Aβ could be associated with Elk1 and Elk2, which are among the TFs we have determined FpClass, over ERK-2.^100^ The most crucial problem here was that the databases that we used recognize the APP as the target protein, not Aβ since it is a peptide fragment. We could not determine the TFs that could directly interact with Aβ fragments. Because the main protein investigated with FpClass software was APP, PPI estimates including all of its regions could be obtained, and it was not known which of these could be associated with Aβ fragments. To solve this problem, we used the database of TRRUST v2 (an expanded reference database of human and mouse transcriptional regulatory interactions. *Nucleic Acids Research* Oct 26, 2017) with the help of the data we obtained from our previous studies. The TRRUST v2 database functions by bringing together studies of related genes from the PubMed database.^101^ Using the genes that we know that their mRNA levels change in response to Aβ1–42 application in our and other previous studies^102−105^ as targets in TRRUST v2 database, we determined TFs related to them. The TFs, which we previously determined to have a possible relationship with APP using FpClass PPI software,^97^ were compared with TFs that play a role in the transcription of genes reported by our group^63,102−104^ and the TFs given in TRRUST database. This comparison is used to determine common TFs. Considering the FpClass software PPI scores and the number of genes whose expression is regulated by Aβ, the possible TFs that Aβ1–42 can work together were determined. Potential TFs determined by combining FpClass and TRRUST data for Aβ1–42 are ETS2, JUN, JUND, SP1, STAT1, STAT3, TBP, SMAD3, SNAl1, NFKB1, RELA, ELK1, ATF4, TFCP2, APC, FOS, FOSL2. Confirming this information, in a recent study, it has been shown the changed expression of JUN and ATF4 TFs in single-soma transcriptomics of tangle-bearing neurons in AD.^106^

The same was done for α-syn. In addition to the studies conducted by other groups showing that α-syn affects the expression of different genes,^107−109^ data belonging to 41 growth factors published by our group in 2020^110^ and 40 inflammation factors prepared for publication were used. Possible TFs determined by combining FpClass and TRRUST data for α-syn are ATF1, ATF2, NFKBIA, RELA, MYC, CTNNB1, CEBPB, FOS, JUN, JUND, IRF3, APEX1, NR3C1, ELK1, HMGA1, HSF1, KLF4, STAT3, SMAD3, EP300, TP53.

## Peptides That Are Localized to Mitochondria and Have the Ability To Bind to mtDNA or RNAs or TFs May Carry Post-Translational Modifications

We think that Aβ1–42 can be translocated into the mitochondria by phosphorylation, just as α-syn can be displaced within the cell as a result of phosphorylation.^111−114^ Aβ has derivatives such as 1–40 and 1–42 as well as post-translationally modified variants. These post-translational modifications include splicing, racemization (optically inactivation), isomerization, pyroglutamination, metal-induced oxidation, and phosphorylations. Modified Aβ derivatives are generally highly toxic and induce aggregation formation as a type of seeding. These variants are present from the early stages of AD.^115^ Phosphorylation is a reversible post-translational modification that can alter the structural and functional properties of proteins. Phosphorylation appears to be an essential step, especially in mechanisms related to protein activity, cell cycle control, gene regulation, learning, and memory.^116,117^ In silico analysis has shown that Aβ can be phosphorylated at certain positions. It has been reported that these phosphorylations can be performed by protein kinase A and Cdc2 in vitro, in culture cells, or in human cerebrospinal fluid.^115,118^ Although recent studies indicate that these phosphorylations stabilize the oligomeric structure of the peptide and increase its toxicity,^119^ it has been shown that serine eight phosphorylation of Aβ 1–42 decreases its toxicity, whereas it increases binding to membrane lipids.^120^ However, no study on subcellular localization of phosphorylated amyloid-β peptides has been found in the literature.

## Conclusion

We suggest that under physiological conditions, Aβ1–42 or other fragments and α-syn may have an essential function on the mitochondrial genome. A detailed review by Doig et al. shows, in particular, that we do not know the true functions of Aβ and its behavior in pathological conditions.^121^ One of these functions may be the direct regulation of mtDNA gene expression. We also suggest that revealing these peptides’ physiological roles in the mitochondria will enable us to understand better the effects of pathological protofibrils or fibril forms of peptides on accumulated mitochondrial damage in the progressive neurodegeneration process. But we need detailed studies to confirm our hypothesis. A recent study suggested that the mitochondrial function determines Aβ release of the cells, and Aβ fluid levels and ratios might serve as biomarkers of mitochondrial integrity.^122^ In our opinion, it seems unlikely that a protein or peptide would make such extreme changes in an organelle that did not function under physiological conditions.

Mitochondrial functions are a key point, especially for postmitotic cells with high energy needs. When we look in terms of neurodegeneration, it is known that mitochondrial dysfunctions occur before the disease symptoms appear. While recent studies put mitochondria at a critical point in terms of aging and neurodegeneration, it has begun to change the belief that the nucleus rules the mitochondria to “mitochondria also govern the nucleus and other organelles”.^123^ If any of these peptides could be shown to be involved in mitochondrial gene expression in some way, it would provide us with two significant pieces of information. First, it will turn out that they may be these peptides participating in the regulation of mtDNA transcription in a healthy cell, enabling the execution of mitochondrial functions. This will lead to a reconsideration of treatment strategies that directly target these two peptides. Second, before the symptoms appear in pathological conditions, the transcripts of mtDNA, which has far fewer genes, can be followed in the early stages of diseases. On the other hand, if the binding sites of these peptides to mtDNA can be determined, there will be certain changes in the understanding of the pathological processes. For example, possible nucleotide changes in these regions may change the binding patterns of monomers under physiological conditions and can lead to mitochondrial dysfunction. Or the protofibrils or fibrils of peptides may lose their binding capacity to their binding sites in a disease state and may result in pathological condition. Both of the conditions may provide new parameters that can be followed in the emergence, progression, and severity of the disease. If these peptides also interact with mitochondrial TFs and alter mtDNA replication and transcription in this way, further work may be required on diagnostic and perhaps therapeutic approaches, especially for mitochondria. In case RNAs that cause imbalance are identified, it will provide a resource for developing RNA aptamers and directing them toward mitochondria-specific treatment strategies. This hypothesis may be expanded for all amyloid forming peptides, but we need detailed studies and different perspectives to answer these questions.
